# Natural products-based: Synthesis and antifungal activity evaluation of novel L-pyroglutamic acid analogues

**DOI:** 10.3389/fpls.2022.1102411

**Published:** 2022-12-22

**Authors:** Likun Ai, Shiqi Fu, Yong Li, Mei Zuo, Wen Huang, Jian Huang, Zhichao Jin, Yang Chen

**Affiliations:** ^1^ State Key Laboratory Breeding Base of Green Pesticide and Agricultural Bioengineering, Key Laboratory of Green Pesticide and Agricultural Bioengineering, Ministry of Education, Guizhou University, Guiyang, China; ^2^ State Key Laboratory of Natural and Biomimetic Drugs, Peking University, Beijing, China; ^3^ College of Pharmacy, Guizhou Medical University, Guiyang, China

**Keywords:** L-pyroglutamic acid, chiral hydroxyl, benzenesulfonyl derivatives, antifungal activity, botanical Pesticides

## Abstract

Botanical pesticides are one of the sources of third-generation pesticides, which have received much attention at home and abroad in recent years due to their degradable and pollution-free advantages in nature. This article explored a concise approach toward synthesizing a series of novel L-pyroglutamic acid analogues from L-hydroxyproline. Furthermore, bioassay studies of these sulfonyl ester derivatives against *Pyricularia oryzae*, *Fusarium graminearum*, *Alternaria brassicae*, *Valsa mali*, and *Alternaria alternariae* showed moderate antifungal activity. For instance, **C08a** and **C08l** provide potential lead agents for controlling *Fusarium graminearum* because of their inhibitory activity.

## Introduction

Nature is a massive library of compounds. Natural plants have given the reputation of molecular manufacturing factories and organic chemists, providing constant inspiration for human drug design. The plant secondary metabolites mainly include organic acids, terpenoids, and alkaloids with specific structures. The effects of these natural products from plants on the target are shown as insecticidal (Zhao et al., 2016; Wang et al., 2018), antibacterial, (Lin et al., 2014), antitumor (Moutevelis-Minakakis et al., 2011), antimalarial (Amoa Onguéné et al., 2013), and other activities. They are considered ideal lead compounds for developing medical or agricultural chemicals and play an increasingly important role in discovering medicine and green pesticides.

Plant-derived drugs and their derivatives are the primary sources of many essential drugs in medicine and pesticides (Harvey, 2008). They are characterized by high selectivity, low toxicity, easy degradation, and less resistance to natural enemies. These outstanding advantages have led pesticide scientists to pay more attention to traditional herbs, which is significant for discovering novel, environmentally friendly, and sustainable plant-derived pesticides to control agricultural diseases (Newman and Cragg, 2020; Atanasov et al., 2021).

**Figure 1:**
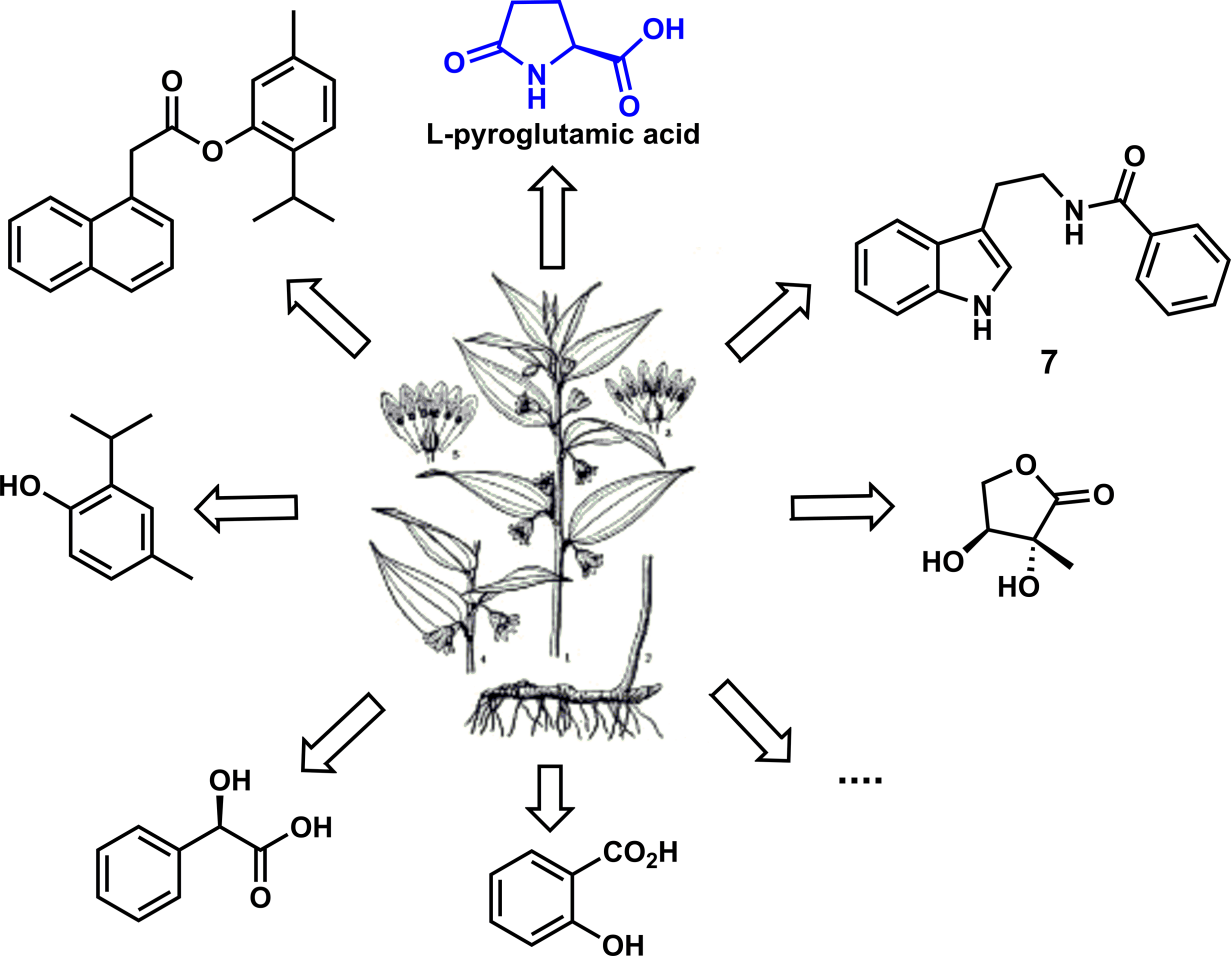
Antifungal compounds from Disporopsis aspersa (HUA) ENGL. ex DIELS.

A typical Chinese herbal medicine, *Disporopsis aspersa* (Hua) Engl. ex Dells from the *Disporopsis HANCE* of Liliaceae perennial herb, has attracted considerable interest owing to its significant biological activities (Wang et al., 2015). For instance, the decoction of rhizomes in *D. aspersa* is primarily prescribed as a tonic for asthenia, night sweats, spermatorrhea, and polyuria. Moreover, it has other effects for treating persistent fever, dry cough, and cancer (Nguyen et al., 2006). Nonetheless, there are a few studies about the antifungal activities of this plant until 2018. As shown in **Figure 1**, Zhang and his colleagues investigated the antifungal activity of the crude extracts from *D. aspersa*. In the biological activity screening, L-pyroglutamic acid showed excellent antifungal activity against *P. infestans* and *P. cubensis* with EC_50_ values of 9.48 and 10.82 μg/ml, respectively, especially the inhibition rate of therapeutic effect, with a prevention rate of 87.1%, which is far more potent than the positive control drug and could be used as a candidate compound of antifungal lead compounds (Zhu et al., 2018).

In 2018, Zhang and his colleagues designed and synthesized a series of derivatives, such as L-pyroglutamate and amide, and systematically evaluated their biological activities. The bioassay and structure–activity relationship (SAR) study showed that most L-pyroglutamate esters had vigorous antibacterial activity (Gang et al., 2018). During the screening process of compound activity, the reaction of L-pyroglutamic acid with 4-chlorophenol to produce ester could effectively improve the antibacterial activity of the compounds. To further study the bioactivity and structure–activity relationship of L-pyroglutamate, the lead compound of botanical drugs was explored.

This study explored a concise approach to synthesize a series of novel L-pyroglutamic acid analogues from L-hydroxyproline, evaluated the antifungal activity of these compounds, and analyzed the structure–activity relationship of L-pyroglutamate.

## Results and discussion

### Design strategies for the skeleton of 4-chiral hydroxyl L-pyroglutamate compounds

**Scheme 1:**
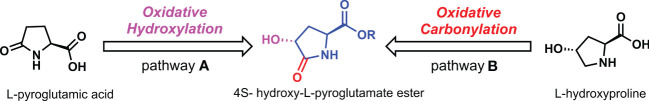
Design strategies for the skeleton of 4-chiral hydroxyl L-pyroglutamate compounds.

Chiral hydroxyl can enhance the biological activity of chiral pesticides, such as uniconazole, diniconazole, and tebuconazole. If a chiral hydroxyl group is assembled in the precursor skeleton molecule of L-pyroglutamate, the bactericidal activity of the molecule can be enhanced without affecting other functional groups. As shown in **Scheme 1**, two methods exist to construct the 4S-hydroxy-L-pyroglutamate ester framework. In pathway A, the chiral hydroxyl was induced to the alpha position of the carbonyl group in L-pyroglutamic acid, as starting material *via* oxidation hydroxylation (Chen et al., 2022; Negi et al., 2022). However, chiral hydroxyl groups have yet to be successfully constructed after many asymmetric oxidation methods have been tried. On the other side of the strategy, the original idea may be realized by preassembling chiral hydroxyl groups onto pyrrole rings of L-hydroxyproline and then oxidative carbonylation of active methylene.

**Scheme 2:**
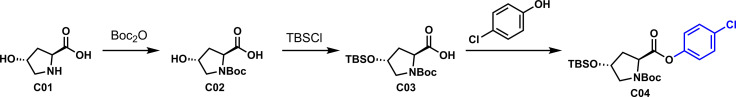
Synthesis of **C04**.

Based on the design idea, L-hydroxyproline was used as the starting material to oxidize carbonyl at the active methylene position. First, the three active functional groups of the reaction starting material must be protected; otherwise, side reactions are easy to occur. As shown in **Scheme 2**, the three functional groups are successively protected by N-Boc amide, OTBS silicon ether, and *p*-chlorophenyl formate to obtain the critical intermediate **C04**. Notably, 4-chlorophenol was introduced to increase the antibacterial activity of our derivatives, inspired by Zhang’s work.

With compound **C04** in hand, the key carbonyl assembly will be investigated. As shown in **Table 1**, we tried to perform oxidative carbonylation *via* TBHP (Hossain and Shyu, 2016), DIB/TBHP (Zhao et al., 2013), and KMnO_4_ (Lai and Lee, 2002). However, we did not get the desired products with the three oxidants (entries 1~3). The subject fell into the darkest, and we searched everywhere for appropriate oxidation methods. Fortunately, Yoshifuji reported a two-phase oxidation reaction using RuO_2_ to convert cyclic A-amino acids to A-amino dicarboxylic acids in 1995 (Yoshifuji and Kaname, 1995). In 2001, Zhang successfully oxidized 4-hydroxyproline methyl ester into 4-hydroxypyroglutamate methyl ester using this method (Zhang et al., 2001). This method is intended to be used for oxidative carbonylation. Encouragingly, compound **C04** was oxidized by RuO_2_·H_2_O in the solvent of EtOAc/H_2_O at room temperature to generate amide **C05** with excellent reactivity in 90% yield (entry 4).

**Table 1:** Study of the oxidative carbonylation.

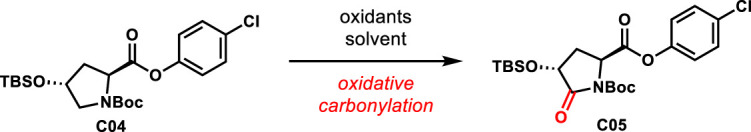

**Table:** 

Entry	Oxidants	Solvent	Yield (%)
1	TBHP	H_2_O	0
2	DIB/TBHP	MeNO_2_	0
3	KMnO_4_	Acetone	0
4	RuO_2_·H_2_O	EtOAc/H_2_O	90

TBHP, butyl hydroperoxide; DIB, 1,4-diphenyl-2,3-benzofuran; RuO_2_·H_2_O (20 mol%).

### Synthesis of L-pyroglutamic acid 4-chiral hydroxyl sulfonyl ester derivatives

Sulfonyl esters are widely used in medicine and pesticides due to their remarkable biological activities. For example, the main active substance thiothiesulfate obtained from garlic extraction and separation not only has the effect of reducing blood lipid (Ackermann et al., 2001; Rahman, 2001) but also has antitumor (Bianchini and Vainio, 2001), antiviral, and a variety of medical activities such as antibacterial, bactericidal, and viricidal (Harris et al., 2001). In the study of pesticide activity, sulfonate compounds were shown to have insecticidal, acaricidal, and bactericidal activities. In addition, sulfonate compounds also have herbicidal and plant growth-regulating effects.

Sulfonyl and sulfonamide groups are important pharmacophore groups in many drugs, and introducing these groups can effectively improve the activity of compounds. In 2011, Zhao reported curcumin benzoyl sulfonate compounds’ synthesis and acaricidal activity (Zhao, 2011). Taking curcumin as the lead, the authors produced a reaction with benzene sulfonyl chloride compounds to synthesize a series of curcumin benzoyl sulfonate derivatives and tested the acaricidal activity. The results showed that compared with curcumin itself, its acaricidal and ovicidal activities were significantly improved.

**Scheme 3:**
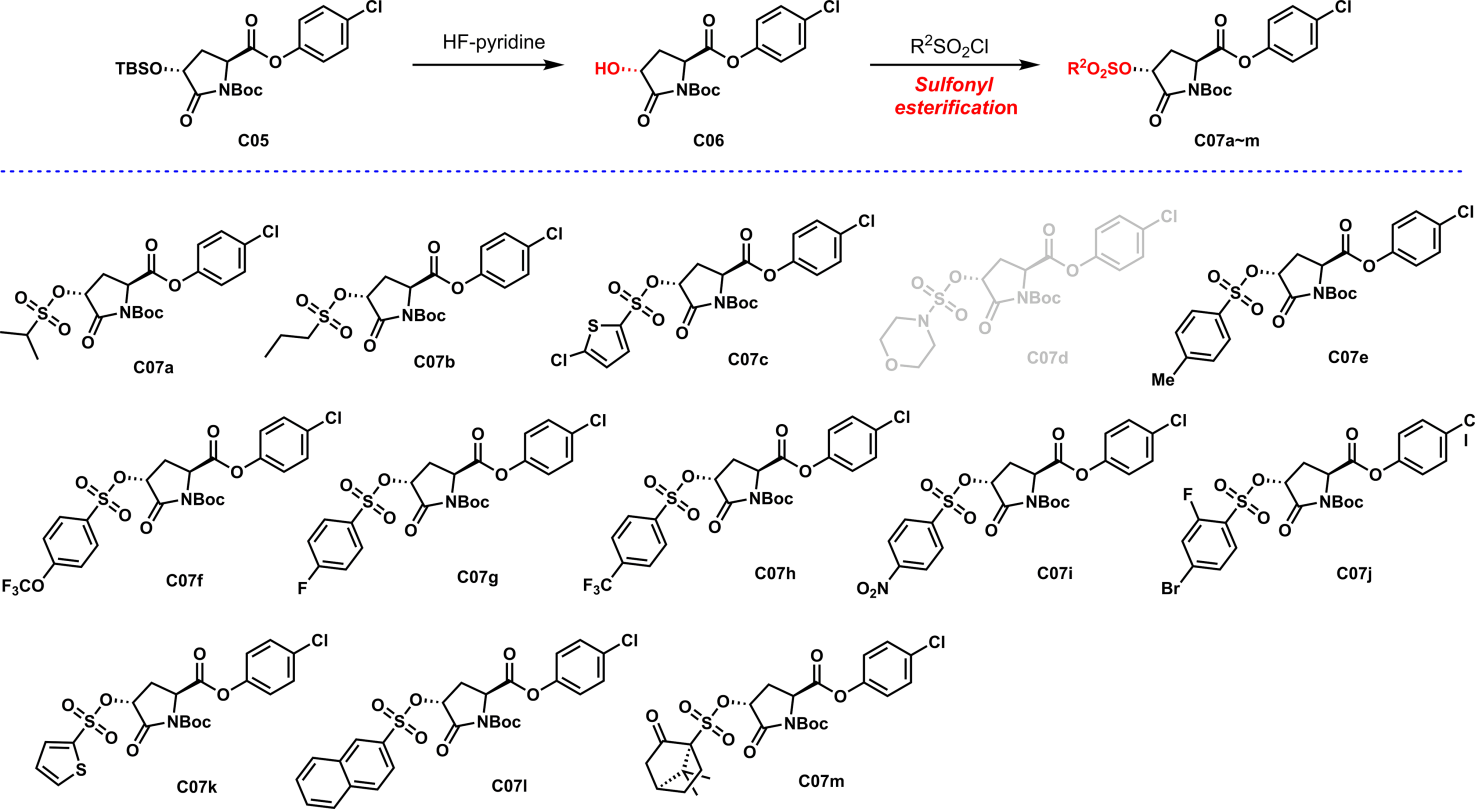
Preparation of sulfonyl esters **C07a~m**.

As shown in **Scheme 3**, sulfonyl esterification was carried out on the chiral hydroxyl group, and different sulfonyl functional groups were introduced to enhance the efficacy. Most sulfonyl groups have a good reaction effect when introduced. Unfortunately, the synthesis of **C07d** showed a poor reaction effect and low yield. The analysis might be due to the presence of the N atom in the sulfonyl group, which increased the density of the electron cloud on the S atom and reduced the overall reactivity, resulting in poor reactivity.

### Deprotection of N-Boc

**Scheme 4:**
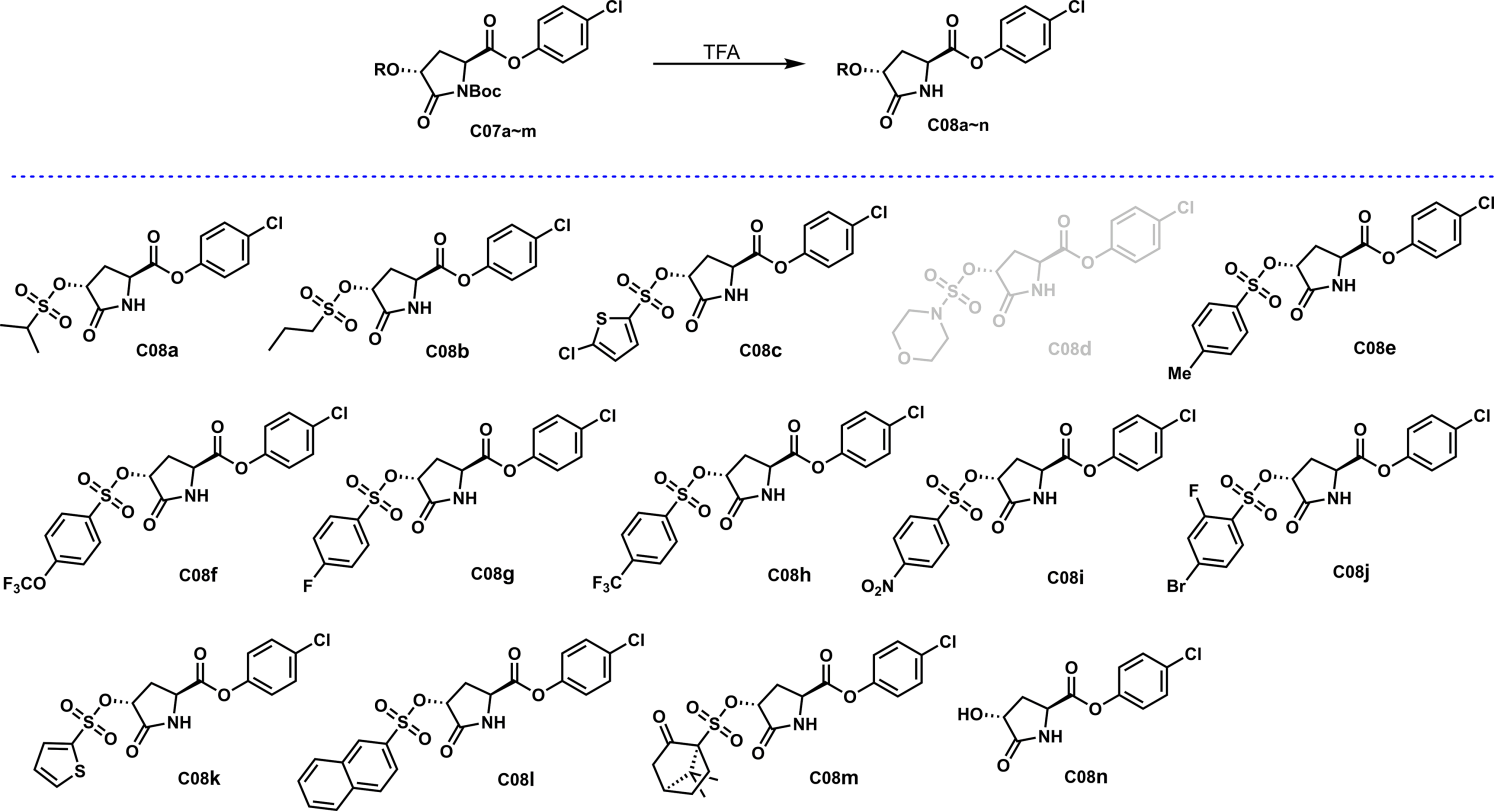
Deprotection of N-Boc.

The amide group, as an essential group in natural products, often appears in a state without other substitutions, which is conducive to the compound’s participation and thus enhances bioactivity. However, the amide group in the **C07** series compounds obtained in this study is connected with the electron-pulling group, which is quite different from the natural products, and may impact the activity. In order to further explore whether the existence of electron-pulling groups on the amide N structure affects the antibacterial activity, as shown in **Scheme 4**, a **C07** series of compounds was successfully removed from N-Boc to obtain **C08a~n** under a solution of trifluoroacetic acid (TFA) in dichloromethane.

### Evaluation of antifungal activity of intermediates and derivatives

In this study, the antibacterial activity of 31 compounds against five species of fungi (*Pyricularia oryzae*, *Fusarium graminearum*, *Alternaria brassicae*, *Valsa mali*, and *Alternaria alternariae*) at 100 μg/ml was evaluated by an approach of inhibiting mycelium growth rate. The preliminary bioactivity test results displayed that most of the 31 target compounds had inhibitory effects on these five fungi. Among them, compounds **C07l~m** and **C08a~n** were more potent than any other against *Fusarium graminearum* and *Valsa mali*, they were superior to the commercial control drug hymexazol and equal to chlorothalonil. It is shown in **Table 2** that the antibacterial activities of **C07l** and **C07m** were equal to those of **C08l** and **C08m**, but the antibacterial activities of other compounds were lower than those of **C08** series compounds, the results indicated that the amide group attached to the Boc group was detrimental to the inhibitory activity. The activities of L-pyroglutamic acid derivatives with alkane chains were slightly higher than those of aromatic compounds. In addition, the introduction of naphthalene (**C08l**) and alkane (**C08a**) on the hydroxyl group could effectively enhance the inhibitory activity of *Fusarium graminearum*. As for the effects of substituents on aromatic compounds, available data exhibited that an electron-withdrawing group played an important role to the enhancement of antibacterial activity.

**Table 2:** The inhibitory rates of the L-pyroglutamic acid derivatives on phytopathogenic fungi (100 μmol/l).

Compounds	Antifungal activities (inhibition %)				
	*BH*	*YC*	*PF*	*XC*	*SD*
CK	0 ( ± 0.9)	0 ( ± 0.8)	0 ( ± 1.3)	0 ( ± 0.6)	0 ( ± 1)
Hymexazol	73.6 ( ± 1.8)	71.4 ( ± 0.8)	37.3 ( ± 1.7)	35.1 ( ± 1.2)	68.9 ( ± 2.1)
Chlorothalonil	35.8 ( ± 0.9)	60.7 ( ± 0.8)	69.3 ( ± 0.6)	72.7 ( ± 0.6)	48.9 ( ± 1)
**C01**	42.6 ( ± 0.9)	36.1 ( ± 0.8)	33.3 ( ± 1.3)	29.1 ( ± 1.2)	28.9 ( ± 1)
**C02**	-	21.8 ( ± 0.8)	25.3 ( ± 0.6)	21.3 ( ± 0.6)	22.2 ( ± 1)
**C03**	0.0( ± 1.8)	28.9 ( ± 0.8)	30.7 ( ± 0.6)	22.6 ( ± 0.6)	28.9 ( ± 1)
**C04**	23.8 ( ± 0.9)	28.9 ( ± 0.8)	30.7 ( ± 0.6)	33.0 ( ± 0.6)	28.9 ( ± 2.1)
**C05**	25.7 ( ± 0.9)	27.1 ( ± 1.7)	41.3 ( ± 0.6)	30.4 ( ± 0.6)	28.9 ( ± 1)
**C06**	37.0 ( ± 0.9)	37.9 ( ± 0.8)	52.0 ( ± 0.6)	51.2 ( ± 0.6)	37.8 ( ± 1)
**C07a**	25.7 ( ± 0.9)	27.1 ( ± 1.7)	40.0 ( ± 1.3)	22.6 ( ± 1.2)	26.7 ( ± 1)
**C07b**	21.9 ( ± 1.8)	25.4 ( ± 0.8)	36.0 ( ± 0.6)	21.3 ( ± 0.6)	26.7 ( ± 1)
**C07c**	-	23.6 ( ± 1.7)	34.7 ( ± 1.9)	21.3 ( ± 0.6)	20 ( ± 2.1)
**C07e**	-	30.7 ( ± 0.8)	41.3 ( ± 0.6)	27.8 ( ± 0.6)	31.1 ( ± 1)
**C07f**	-	25.4 ( ± 0.8)	28.0 ( ± 1.3)	21.3 ( ± 0.6)	28.9 ( ± 1)
**C07g**	-	25.4 ( ± 0.8)	36.0 ( ± 0.6)	34.3 ( ± 0.6)	33.3 ( ± 2.1)
**C07h**	-	27.1 ( ± 1.7)	36.0 ( ± 0.6)	-	26.7 ( ± 1)
**C07i**	29.4 ( ± 10.3)	23.6 ( ± 2.5)	22.7 ( ± 1.9)	21.3 ( ± 0.6)	22.2 ( ± 1)
**C07j**	21.9 ( ± 0.9)	30.7 ( ± 0.8)	44.0 ( ± 0.6)	29.1 ( ± 1.2)	31.1 ( ± 2.1)
**C07k**	20.0 ( ± 0.9)	28.9 ( ± 0.8)	37.3 ( ± 1.3)	23.9 ( ± 0.6)	33.3 ( ± 2.1)
**C07l**	38.9 ( ± 0.9)	43.2 ( ± 2.5)	49.3 ( ± 0.6)	60.3 ( ± 0.6)	44.4 ( ± 1)
**C07m**	37.0 ( ± 1.8)	45.0 ( ± 0.8)	48.0 ( ± 0.6)	60.3 ( ± 1.8)	44.4 ( ± 1)
**C08a**	37.0 ( ± 0.9)	43.2 ( ± 1.7)	44.0 ( ± 0.6)	61.6 ( ± 1.2)	42.2 ( ± 1)
**C08b**	33.2 ( ± 0.9)	39.6 ( ± 0.8)	46.7 ( ± 1.9)	59.0 ( ± 0.6)	42.2 ( ± 1)
**C08c**	37.0 ( ± 0.9)	45.0 ( ± 1.7)	40.0 ( ± 0.6)	55.1 ( ± 1.8)	42.2 ( ± 1)
**C08e**	35.1 ( ± 0.9)	43.2 ( ± 0.8)	56.0 ( ± 0.6)	49.9 ( ± 1.2)	46.7 ( ± 1)
**C08f**	35.1 ( ± 2.7)	43.2 ( ± 0.8)	52.0 ( ± 1.3)	46.0 ( ± 0.6)	46.7 ( ± 1)
**C08g**	33.2 ( ± 0.9)	41.4 ( ± 0.8)	42.7 ( ± 0.6)	53.8 ( ± 0.6)	42.2 ( ± 1)
**C08h**	38.9 ( ± 3.6)	41.4 ( ± 1.7)	46.7 ( ± 0.6)	57.7 ( ± 0.6)	40.0 ( ± 1)
**C08i**	37.0 ( ± 2.4)	45.0 ( ± 0.8)	46.7 ( ± 0.6)	52.5 ( ± 0.6)	44.4 ( ± 1)
**C08j**	33.2 ( ± 0.9)	43.2 ( ± 1.7)	45.3 ( ± 1.3)	52.5 ( ± 0.6)	44.4 ( ± 1)
**C08k**	33.2 ( ± 0.9)	41.4 ( ± 0.8)	41.3 ( ± 0.6)	59.0 ( ± 0.6)	35.6 ( ± 1)
**C08l**	35.1 ( ± 1.8)	43.2 ( ± 0.8)	46.7 ( ± 0.6)	61.6 ( ± 0.6)	44.4 ( ± 2.1)
**C08m**	35.1 ( ± 0.9)	46.8 ( ± 0.8)	48.0 ( ± 1.3)	60.3 ( ± 0.6)	44.4 ( ± 1)
**C08n**	37.0 ( ± 5.3)	45.0 ( ± 0.8)	48.0 ( ± 1.3)	60.3 ( ± 0.6)	44.4 ( ± 1)

Ribociclib was used as positive control. BH: Alternaria brassicae. YC: Alternaria alternariae. PF: Valsa mali. XC: Fusarium graminearum. SD: Pyricularia oryzae.

According to the above results, the structure–activity relationship (SAR) could be done. All **C08** series compounds showed significant antifungal activities against *Fusarium graminearum* (**Table 2**). In particular, the activity of the compounds with naphthalene was more potent than those with phenyls, and the electron-withdrawing groups on the aromatic ring facilitated the inhibitory effects of **C08e~k**. In addition, the introduction of 4-chlorophenol into compound **C04** was beneficial to the antibacterial activity against the five fungi.

## Materials and methods

### Equipment and materials

All reactions were performed in flame-dried glassware under a nitrogen atmosphere. Solvents were distilled prior to use. Reagents were used as purchased from Aladdin, Macklin, Innochem, or TLC unless otherwise noted. Chromatographic separations were performed using a silica gel, AR, 200–300 mesh. ^1^H and ^13^C NMR spectra were obtained on Bruker 400 MHz NMR and JNM-ECZR 500 MHz NMR instruments using CDCl_3_ and DMSO as the solvent, which were provided by the School of State Key Laboratory Breeding Base of Green Pesticide and Agricultural Bioengineering, Key Laboratory of Green Pesticide and Agricultural Bioengineering, Ministry of Education, Guizhou University. Optical rotations were obtained on an InsMark digital polarimeter using a sodium (589 nm, D line) lamp and are reported as follows: ${\left[\text{α}\right]}_{\text{λ}}^{\text{T }{}^{\circ}\text{C}}$ (c = g/100 ml, solvent). TLC analysis was visualized using UV and phosphomolybdic acid stains. High-resolution mass spectra were obtained using Q Exactive. All spectral data obtained for new compounds are reported here.

The biological reagents used were glucose, AGAR, and streptomycin. In addition, potatoes were bought from supermarkets.

### Synthetic procedures for the key intermediate C06

**Scheme 5:**
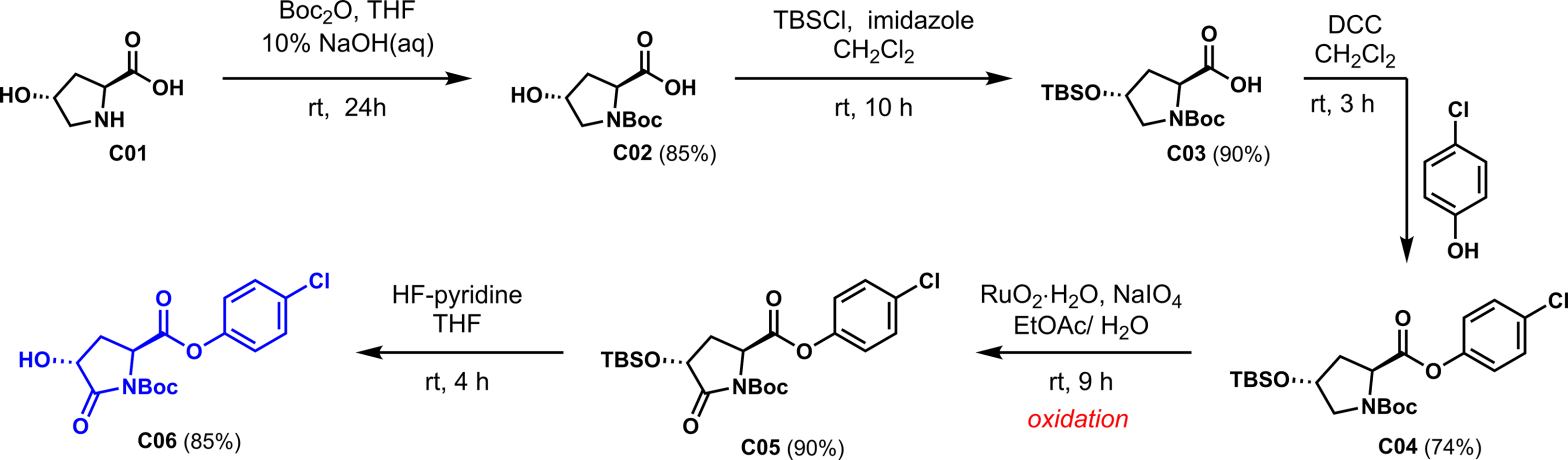
Synthesis of L-pyroglutamic acid 4-chiral hydroxy derivative via linear strategy.

The synthetic procedures for the key intermediate **C06** from L-hydroxyproline are shown in **Scheme 5**.

To a solution of L-hydroxyproline **C01** [20.0 g, 152 mmol, ${\left[\text{α}\right]}_{D}^{25}=+89.7\left(\text{c}0.1,{\text{H}}_{2}\text{O})\right]$ in H_2_O (76 ml) was added a solution of 10% NaOH (aqueous, 60 ml, 167 mmol). The resulting solution was stirred for 2 h followed by adding a solution of Boc_2_O (36.6 g, 167 mmol) in THF (152 ml) in one portion *via* a syringe. The mixture was stirred for 24 h at room temperature (rt), and the reaction was quenched by addition of 10% KHSO_4_ solution in water to adjust pH = 4~5, and the organic layer was separated. The aqueous layer was further extracted with EtOAc (100 ml × 3). The combined organic extracts were washed with the saturated solution of NaCl (50 ml × 1), dried over anhydrous Na_2_SO_4_, filtered, and concentrated *in vacuo* to afford **C02** as white solid (30.0 g, 85%) and was used directly in the next step without further purification. Mp = 117.8~118.6 °C. R*_f_* = 0.3 (100% MeOH/CH_2_Cl_2_). ^1^H NMR (400 MHz, DMSO-*d_6_*, 25°C, *mixture of rotamers*) *δ* 12.49 (*br* s, 1H), 5.04 (*br* s, 1H), 4.25~4.22 (m, 1H), 4.11 (td, *J* = 7.9, 2.4 Hz, 1H), 3.33~3.41 (m, 1H), 3.28~3.20 (m, 1H), 2.05~2.14 (m, 1H), 1.84~1.92 (m, 1H), 1.36 (d, *J* = 18.8 Hz, 9H). ^13^C NMR (100 MHz, DMSO-*d_6_*, 25°C, *mixture of rotamers*) *δ* 174.5, 174.0, 153.9, 153.3, 78.9, 78.9, 68.6, 67.9, 57.8, 57.5, 54.7, 54.4, 28.2, 28.0. HRMS (ESI+): m/z calculated for C_10_H_17_NO_5_ [M+Na]^+^ 254.1106, found 254.0995.

To a solution of **C02** (5.0 g, 22 mmol) in CH_2_Cl_2_ (45 ml) were added *t*-butylchlorodimethylsilane (7.2 g, 48 mmol) and imidazole (6.5 g, 95 mmol). The mixture was stirred for 10 h at room temperature, and the reaction was quenched by addition of 1 M HCl solution to adjust pH = 4~5. The organic layers were separated, and the aqueous layer was extracted with CH_2_Cl_2_ (50 ml × 3). The combined organic extracts were washed with the saturated solution of NaCl (50 ml × 1), dried over anhydrous NaSO_4_, filtered, and concentrated *in vacuo*. The residue was subjected to silica gel chromatography (petroleum ether: EtOAc = 3:1) to afford **C03** as colorless oil (6.7 g, 90%). R*_f_* = 0.3 (30% EtOAc/petroleum ether). ^1^H NMR (500 MHz, CDCl_3_, 25°C, *mixture of rotamers*) *δ* 6.42 (*br* s, 1H), 4.46~4.30 (m, 2H), 3.61~3.45 (m, 1H), 3.41~3.32 (m, 1H), 2.28~2.02 (m, 2H), 1.45 (d, *J* = 31.4 Hz, 9H), 0.86 (s, 9H), 0.06 (d, *J* = 3.5 Hz, 6H). ^13^C NMR (100 MHz, CDCl_3_, 25°C, *mixture of rotamers*) *δ* 178.3, 175.3, 156.6, 154.1, 81.5, 80.7, 70.1, 69.8, 58.1, 55.0, 54.7, 39.9, 38.0, 31.2, 28.5, 28.3, 25.8, 25.77, 25.7, 18.1. HRMS (ESI+): m/z calculated for C_16_H_31_NO_5_Si [M+Na]^+^ 368.2971, found 368. 3153.

To a solution of **C03** (4.2 g, 12 mmol) in CH_2_Cl_2_ (40 ml) were added DCC (3.0 g, 15 mmol) and 4-chlorophenol (1.9 g, 15 mmol). The mixture was stirred for 3 h at room temperature. CH_2_Cl_2_ was added to dilute and filter solids. The organic layer was concentrated *in vacuo*. The residue was subjected to silica gel chromatography (petroleum ether: EtOAc = 10:1) to afford **C04** as colorless solid (4.1 g, 74%). Mp = 56~59.8 °C. R*_f_ =* 0.3 (10% EtOAc/petroleum ether). ^1^H NMR (500 MHz, CDCl_3_, 25°C, *mixture of rotamers*) *δ* 7.35~7.29 (m, 2H), 7.07~7.02 (m, 2H), 4.61~4.48 (m, 1H), 4.47~4.45 (m, 1H), 3.65~3.58 (m, 1H), 3.51~3.35 (m, 1H), 2.37~2.26 (m, 1H), 2.21~2.11 (m, 1H), 1.45 (d, *J* = 12.7 Hz, 9H), 0.87 (s, 9H), 0.07 (s, 6H). ^13^C NMR (100 MHz, CDCl_3_, 25°C, *mixture of rotamers*) *δ* 171.6, 171.5, 154.7, 153.9, 149.3, 149.1, 131.4, 131.3, 129.6, 129.5, 123.0, 122.5, 80.6, 80.4, 70.6, 69.8, 58.3, 58.0, 55.1, 54.8, 40.1, 39.0, 35.0, 28.5, 28.4, 25.8, 25.8, 25.5, 24.8, 18.1, 18.0. HRMS (ESI+): m/z calculated for C_22_H_34_ClNO_5_Si [M+Na]^+^ 478.1894, found 478.1779.

To a solution of NaIO_4_ (3.8 g, 17 mmol) in H_2_O (30 ml) was added RuO_2_·H_2_O (0.13 g, 1 mmol) at room temperature. The resulting green yellow solution was stirred for 2 h followed by addition of **C04** (2.0 g, 4 mmol) in EtOAc (15 ml) in one portion *via* a syringe. Additional aliquots of NaIO_4_ were added to maintain a yellow-colored solution during the reaction. The mixture was stirred for 9 h at room temperature, and EtOAc (100 ml) to dilution reaction. The reaction was quenched by addition of sat. aq. Na_2_S_2_O_3_, which immediately resulted in the precipitation of Ru black. The organic layers were separated, and the organic extract was filtered through a pad of Celite. The filtrate was then washed with the saturated solution of NaCl (50 ml × 1), dried over anhydrous Na_2_SO_4_, and evaporated under reduced pressure. The resulting residue was subjected to silica gel chromatography (petroleum ether: EtOAc = 10:1) to afford **C05** as a white solid (1.85 g, 90%). Mp = 73.7~76.9°C. R*_f_* = 0.33 (10% EtOAc/petroleum ether). ^1^H NMR (400 MHz, CDCl_3_, 25°C, *mixture of rotamers*) *δ* 7.20~7.16 (m, 2H), 6.89~6.85 (m, 2H), 4.60 (dd, *J* = 9.8, 1.7 Hz, 1H), 4.31 (dd, *J* = 10.0, 8.2 Hz, 1H), 2.36~2.30 (m, 1H), 2.19~2.11 (m, 1H), 1.34 (s, 9H), 0.72 (s, 9H), 0.05 (d, 6H). ^13^C NMR (100 MHz, CDCl_3_, 25°C, *mixture of rotamers*) *δ* 171.6, 169.7, 149.7, 148.7, 131.9, 129.7, 122.4, 84.4, 69.7, 55.2, 31.8, 28.0, 25.7, 18.2. HRMS (ESI+): m/z calculated for C_22_H_32_ClNO_6_Si [M+Na]^+^ 482.1687, found 492. 1571.

To a solution of **C05** (1.8 g, 4 mmol) in THF (40 ml) at room temperature was added hydrogen fluoride (70% in HF, 3.1 ml, 24 mmol). The mixture was stirred for 4 h at room temperature, and the reaction was quenched by addition of solid NaHCO_3_ filtered and concentrated *in vacuo*. The residue was subjected to silica gel chromatography (petroleum ether: EtOAc = 3:1) to afford **C06** as white solid (1.2 g, 85%). Mp = 120~121 °C. ${\left[\text{α}\right]}_{D}^{25}=+92.3\;\left(\text{c}0:1,{\text{CHCl}}_{3}\right)$ . R*_f_* = 0.4 (100% EtOAc/petroleum ether). ^1^H NMR (400 MHz, CDCl_3_, 25°C, *mixture of rotamers*) *δ* 7.38~7.35 (m, 2H), 7.09~7.05 (m, 2H), 4.84 (dd, *J* = 9.9, 1.2 Hz, 1H), 4.55 (dd, *J* = 10.8, 8.4 Hz, 1H), 3.05~2.97 (m, 1H), 2.60~2.55 (m, 1H), 1.54 (s, 9H). ^13^C NMR (100 MHz, CDCl_3_, 25°C, *mixture of rotamers*) *δ* 200.9, 173.4, 169.2, 149.0, 148.6, 132.0, 129.8, 122.3 84.82, 68.7, 55.5, 30.6, 27.9. HRMS (ESI+): m/z calculated for C_16_H_18_ClNO_6_ [M+Na]^+^ 378.0822, found 378.3245.

### Synthetic procedures for L-pyroglutamic acid 4-chiral hydroxyl sulfonyl ester derivatives

To a solution of **C06** (100 mg, 0.28 mmol) in CH_2_Cl_2_ (1.0 ml) were added DMAP (0.03 mmol) and Et_3_N (45 mg, 0.42 mmol). The resulting solution was stirred for 10 min followed by addition of sulfonyl chloride (0.42 mmol) in CH_2_Cl_2_ (0.5 ml) in one portion *via* a syringe. The reaction mixture was stirred for 1~4 h at room temperature, and the reaction was quenched by addition of sat. aq. NH_4_Cl. The organic layers were separated and the aqueous layer was extracted with CH_2_Cl_2_ (2 ml × 3), and the combined organic extracts were washed with the saturated solution of NaCl (10 ml × 1) and dried over anhydrous Na_2_SO_4_. The residue was subjected to silica gel chromatography (petroleum ether: EtOAc = 10:1) to afford **C07a~m**.

### Synthetic procedures for deprotection of N-Boc

TFA (2.2 equiv) was added to sulfonyl esters **C07a~m** (1.0 equiv) in CH_2_Cl_2_ (0.25 M), and the mixture was stirred at room temperature for 0.5~2 h. The reaction was quenched with saturated NaHCO_3_ aqueous solution, and the mixture was separated through a separating funnel. The aqueous phase was extracted by CH_2_Cl_2_ (×3) and dried with anhydrous Na_2_SO_4_. After removal solvent by reduce pressure, the crude residue was purified using silica gel flash column chromatography [eluent: EtOAc/petroleum ether] to give compound **C08a~n**.

### Biological assays: the antifungal activity assay

*In vitro* antifungal activity: L-pyroglutamic acid analogues were screened *in vitro* for their antifungal activities against five phytopathogenic fungi by poisoned food technique. Five phytopathogenic fungi, namely, *Pyricularia oryzae* (SD), *Fusarium graminearum* (XC), *Alternaria brassicae* (BH), *Valsa mali* (PF), and *Alternaria alternariae* (YC), were used for the assays. Potato dextrose agar (PDA) medium was prepared in the flasks and sterilized. The target compounds were dissolved in acetone before mixing with PDA, and the concentration of the test compounds in the medium was fixed at 100 μg/ml. The medium was then poured into sterilized Petri dishes. All types of fungi were incubated in PDA at 27 ± 1°C for 4 days to get new mycelia for the antifungal assays, and a mycelium disk of approximately 4 mm in diameter cut from the culture medium was picked up with a sterilized inoculation needle and inoculated in the center of the PDA Petri dishes. The inoculated Petri dishes were incubated at 27 ± 1°C for 4 days. Acetone without any compounds mixed with PDA was served as a negative control, whereas hymexazol and chlorothalonil, two commercial agricultural fungicides, were used as positive controls. For each treatment, three replicates were conducted. The radial growths of the fungal colonies were measured, and data were statistically analyzed. The inhibitory effects of the test compounds on these fungi *in vitro* were calculated by the following formula: Inhibition rate (%) = (*C*-*T*) × 100/(*C*-4 mm), where *C* represents the diameter of fungi growth on untreated PDA and *T* represents the diameter of fungi on treated PDA. Statistical analysis was processed by the SPSS 21.0 (SPSS Inc., Chicago, USA) software.

## Conclusion

In summary, a novel method of oxycarbonylation was used to construct the target skeleton successfully. At the same time, the introduction of the chiral hydroxyl group increased the reaction site, and a series of L-pyroglutamic acid derivatives were synthesized through diversification and their antifungal activities were evaluated. According to the bioassay results, most L-pyroglutamic acid derivatives showed good antibacterial activity against *Fusarium graminearum*, which are superior to commercially available hymexazol compounds **C08a** and **C08l** showing the best activity and being similar to commercially available chlorothalonil. To the best of our knowledge, this is the first report on antifungal properties of chiral 4-hydroxyl L-pyroglutamic acid derivatives.

## Data availability statement

The original contributions presented in the study are included in the article/Supplementary Material. Further inquiries can be directed to the corresponding author.

## Author contributions

YC designed the experiment. YC and LA wrote the manuscript. SF, MZ, and WH prepared all the derivatives and determined the structure *via* spectra. YL and JH performed antifungal activity experiment and analyzed the data. YC and ZJ supervised the entire project. All authors have read and approved the manuscript.
