# Supplementary material for: Natural products-based: Synthesis and antifungal activity evaluation of novel L-pyroglutamic acid analogues
Source: Front Plant Sci. 2022 Dec 22;13:1102411. doi: 10.3389/fpls.2022.1102411 (PMC9815151; doi:10.3389/fpls.2022.1102411)
Supplement: Supplementary file 1 [file DataSheet_1.docx]

**Natural Products-Based: Synthesis and Antifungal Activity Evaluation of Novel L-pyroglutamic Acid Analogues**

Likun Ai^1^, Shiqi Fu^1^, Yong Li^3^, Mei Zuo^1^, Wen Huang^1^, Jian Huang^1^, Zhichao Jin^1^, Yang Chen^1, 2^*

^1^State Key Laboratory Breeding Base of Green Pesticide and Agricultural Bioengineering, Key Laboratory of Green Pesticide and Agricultural Bioengineering, Ministry of Education, Guizhou University, Guiyang 550025, China

^2^State Key Laboratory of Natural and Biomimetic Drugs, Peking University, Beijing 100191, China

^3^College of Pharmacy, Guizhou Medical University, Guiyang 550004, China

***Corresponding author**

Dr. Yang Chen, E-mail: [ychen1@gzu.edu.cn](mailto:ychen1@gzu.edu.cn)

**SUPPLEMENTARY INFORMATION**

**Supplementary Methods**

The detailed characterization data of intermediates and target compounds

**Supplementary Results**

Figures. S1. The ^1^H NMR and ^13^C NMR spectra of intermediates and target compounds

**Supplementary Methods**

The detailed characterization data of intermediates and target compounds

To a solution of L-hydroxyproline **C01** (20.0 g, 152 mmol) in H_2_O (76 mL) was added a solution of 10% NaOH (aqueous, 60 mL, 167 mmol). The resulting solution was stirred for 2 h followed by adding a solution of Boc_2_O (36.6 g, 167 mmol) in THF (152 mL) in one portion via syringe. The mixture was stirred for 24 h at room temperature (rt), and the reaction was quenched by addition of 10% KHSO_4_ solution in water to adjust pH = 4~5, and the organic layer was separated. The aqueous layer was further extracted with EtOAc (100 mL×3). The combined organic extracts were washed with the saturated solution of NaCl (50 mL×1), dried over anhydrous Na_2_SO_4_, filtered and concentrated *in vacuo* to afford **C02** as white solid (30.0 g, 85%) and was used directly in the next step without further purification. Mp. =117.8~118.6 °C. R*_f =_* 0.3 (100% MeOH/ CH_2_Cl_2_). ^1^H NMR (400 MHz, DMSO-*d_6_*, 25 *°*C, *mixture of rotamers*) *δ* 12.49 (*br* s, 1H), 5.04 (*br* s, 1H), 4.25~4.22 (m, 1H), 4.11 (td, *J* = 7.9, 2.4 Hz, 1H), 3.33~3.41 (m, 1H), 3.28 ~ 3.20 (m, 1H), 2.05~2.14 (m, 1H), 1.84~1.92 (m, 1H), 1.36 (d, *J* = 18.8 Hz, 9H). ^13^C NMR (100 MHz, DMSO-*d_6_*, 25 *°*C, *mixture of rotamers*) *δ* 174.5, 174.0, 153.9, 153.3, 78.9, 78.9, 68.6, 67.9, 57.8, 57.5, 54.7, 54.4, 28.2, 28.0. HRMS (ESI+): m/z calculated for C_10_H_17_NO_5_ [M+Na] ^+^ 254.1106, found: 254.0995.

To a solution of **C02** (5.0 g, 22 mmol) in CH_2_Cl_2_ (45 mL), was added *t*-butylchlorodimethyl silane (TBSCl, 7.2 g, 48 mmol) and imidazole (6.5 g, 95 mmol). The mixture was stirred for 10 h at room temperature, and the reaction was quenched by addition of 1 M HCl solution to adjust pH 4~5. The organic layer was separated and the aqueous layer was extracted with CH_2_Cl_2_ (50 mL×3). The combined organic extracts were washed with the saturated solution of NaCl (50 mL×1), dried over anhydrous NaSO_4_, filtered and concentrated *in vacuo*. The residue was subjected to silica gel chromatography (petroleum ether: EtOAc = 3:1) to afford **C03** as colorless oil (6.7 g, 90%). R*_f =_* 0.3 (30% EtOAc/ petroleum ether). ^1^H NMR (500 MHz, CDCl_3_, 25 *°*C, *mixture of rotamers*) *δ* 6.42 (*br* s, 1H), 4.46~4.30 (m, 2H), 3.61~ 3.45 (m, 1H), 3.41~3.32 (m, 1H), 2.28~2.02 (m, 2H), 1.45 (d, *J* = 31.4 Hz, 9H), 0.86 (s, 9H), 0.06 (d, *J* = 3.5 Hz, 6H). ^13^C NMR (100 MHz, CDCl_3_, 25 *°*C, *mixture of rotamers*) *δ* 178.3, 175.3, 156.6, 154.1, 81.5, 80.7, 70.1, 69.8, 58.1, 55.0, 54.7, 39.9, 38.0, 31.2, 28.5, 28.3, 25.8, 25.77, 25.7, 18.1. HRMS (ESI+): m/z calculated for C_16_H_31_NO_5_Si [M+Na] ^+^ 368.2971, found 368.3153.

To a solution of **C03** (4.2 g, 12 mmol) in CH_2_Cl_2_ (40 mL), was added DCC (3.0 g, 15 mmol) and 4-Chlorophenol (**3**, 1.9 g, 15 mmol). The mixture was stirred for 3 h at room temperature. Added CH_2_Cl_2_ to dilute and filter. The organic layer is concentrated *in vacuo*. The residue was subjected to silica gel chromatography (petroleum ether: EtOAc=10:1) to afford **C04** as colorless solid (4.1 g, 74%). Mp. = 56.0~59.8 °C. R*_f =_* 0.3 (10% EtOAc/ petroleum ether). ^1^H NMR (500 MHz, CDCl_3_, 25 *°*C, *mixture of rotamers*) *δ* 7.35~7.29 (m, 2H), 7.07~7.02 (m, 2H), 4.61~ 4.48 (m, 1H), 4.47~ 4.45 (m, 1H), 3.65~3.58 (m, 1H), 3.51~3.35 (m, 1H), 2.37~2.26 (m, 1H), 2.21~2.11 (m, 1H), 1.45 (d, *J* = 12.7 Hz, 9H), 0.87 (s, 9H), 0.07 (s, 6H). ^13^C NMR (100 MHz, CDCl_3_, 25 *°*C, *mixture of rotamers*) *δ* 171.6, 171.5, 154.7, 153.9, 149.3, 149.1, 131.4, 131.3, 129.6, 129.5, 123.0, 122.5, 80.6, 80.4, 70.6, 69.8, 58.3, 58.0, 55.1, 54.8, 40.1, 39.0, 35.0, 28.5, 28.4, 25.8, 25.8, 25.5, 24.8, 18.1, 18.0. HRMS (ESI+): m/z calculated for C_22_H_34_ClNO_5_Si [M+Na] ^+^, 478.1894, found 478.1779.

To a solution of NaIO_4_ (3.8 g, 17 mmol) in H_2_O (30 mL) was added RuO_2_·H_2_O (0.13 g, 1 mmol) under the room temperature. The resulting green yellow solution was stirred for 2 h followed by addition of **C04** (2.0 g, 4 mmol) in EtOAc (15 mL) in one portion via syringe. Additional aliquots of NaIO_4_ were added to maintain a yellow-colored solution during the reaction. The mixture was stirred for 9 h at room temperature, and EtOAc (100 mL) to dilution reaction. The reaction was quenched by addition of sat. aq. Na_2_S_2_O_3_, which immediately resulted in the precipitation of Ru black. The organic layers were separated and the organic extract was filtered through a pad of Celite. The filtrate was then washed with the saturated solution of NaCl (50 mL×1), dried over anhydrous Na_2_SO_4_ and evaporated under reduced pressure. The resulting residue was subjected to silica gel chromatography (petroleum ether: EtOAc = 10:1) to afford **C05** as a white solid (1.85 g, 90%). Mp. =73.7~76.9 °C. R*_f_* = 0.33 (10% EtOAc/ petroleum ether). ^1^H NMR (400 MHz, CDCl_3_, 25 *°*C, *mixture of rotamers*) *δ* 7.20~7.16 (m, 2H), 6.89~6.85 (m, 2H), 4.60 (dd, *J* = 9.8, 1.7 Hz, 1H), 4.31 (dd, *J* = 10.0, 8.2 Hz, 1H), 2.36~2.30 (m, 1H), 2.19~2.11 (m, 1H), 1.34 (s, 9H), 0.72 (s, 9H), 0.05 (d, 6H). ^13^C NMR (100 MHz, CDCl_3_, 25 *°*C, *mixture of rotamers*) *δ* 171.6, 169.7, 149.7, 148.7, 131.9, 129.7, 122.4, 84.4, 69.7, 55.2, 31.8, 28.0, 25.7, 18.2. HRMS (ESI+): m/z calculated for C_22_H_32_ClNO_6_Si [M+Na] ^+^ 482.1687, found 492.1571.

To a solution of **C05** (1.8 g, 4 mmol) in THF (40 mL) at room temperature was added HF⋅pyr. (70% in HF, 3.1 mL, 24 mmol). The mixture was stirred for 4 h at room temperature, and the reaction was quenched by addition of solid NaHCO_3_ filtered and concentrated *in vacuo*. The residue was subjected to silica gel chromatography (petroleum ether: EtOAc = 3:1) to afford **C06** as white solid (1.2 g, 85%). Mp. =120~121 °C. ${[\alpha]}_{D}^{25}$ = + 92.3 (c 0.1, CHCl_3_). R*_f_* =0.4 (100% EtOAc/ petroleum ether). ^1^H NMR (400 MHz, CDCl_3_, 25 *°*C, *mixture of rotamers*) *δ* 7.38~ 7.35 (m, 2H), 7.09~7.05 (m, 2H), 4.84 (dd, *J* = 9.9, 1.2 Hz, 1H), 4.55 (dd, *J* = 10.8, 8.4 Hz, 1H), 3.05~ 2.97 (m, 1H), 2.60~ 2.55 (m, 1H), 1.54 (s, 9H). ^13^C NMR (100 MHz, CDCl_3_, 25 *°*C, *mixture of rotamers*) *δ* 200.9, 173.4, 169.2, 149.0, 148.6, 132.0, 129.8, 122.3 84.82, 68.7, 55.5, 30.6, 27.9. HRMS (ESI+): m/z calculated for C_16_H_18_ClNO_6_ [M+Na] ^+^ 378.0822, found 378.3245.

**General Procedure**: To a solution of **C06** (100 mg, 0.28 mmol) in CH_2_Cl_2_ (1 ml), was added DMAP (0.03 mmol) and Et_3_N (45 mg, 0.42 mmol). The resulting solution was stirred for 10 min followed by addition of sulfonyl chloride (0.42 mmol) in CH_2_Cl_2_ (0.5 mL) in one portion via syringe. The reaction mixture was stirred for 1~4 h at room temperature, and the reaction was quenched by addition of sat. aq. NH_4_Cl. The organic layers were separated and the aqueous layer was extracted with CH_2_Cl_2_ (2 mL×3), and combined organic extracts were washed with the saturated solution of NaCl (10 mL×1), dried over anhydrous Na_2_SO_4_. The residue was subjected to silica gel chromatography (petroleum ether: EtOAc = 10:1) to afford **C07a~m**.

**C07a**: 85 mg (85%). slight yellow oil. ^1^H NMR (400 MHz, CDCl_3_, 25 *°*C, *mixture of rotamers*) *δ* 7.38 (d, *J* = 8.9 Hz, 2H), 7.07 (d, *J* = 8.9 Hz, 2H), 5.26~ 5.31 (dd, *J* = 10.3, 8.7 Hz, 1H), 4.87 (dd, *J* = 10.0, 1.4 Hz, 1H), 3.68~ 3.61 (m, 1H), 2.85~ 2.79 (m, 1H), 2.65~ 2.57 (m, 1H), 1.54 (s, 9H), 1.50 (t, *J* = 6.5 Hz, 6H). ^13^C NMR (100 MHz, CDCl_3_, 25 *°*C, *mixture of rotamers*) *δ* 168.9, 167.1, 148.9, 148.5, 132.2, 129.9, 122.4, 85.6, 74.3, 55.4, 54.4, 29.6, 28.0, 17.0, 16.5. HRMS (ESI+): m/z calculated for C_19_H_24_ClNO_8_S [M+Na] ^+^ 484.9100, found 484.0797.

**C07b**: 110 mg (85%). slight yellow oil. ^1^H NMR (400 MHz, CDCl_3_), 25 *°*C, *mixture of rotamers*) *δ* 7.39~ 7.35 (m, 2H), 7.09~7.04 (m, 2H), 5.35 (dd, *J* = 10.4, 8.8 Hz, 1H), 4.87 (dd, *J* = 10.0, 1.4 Hz, 1H), 3.43~ 3.30 (m, 2H), 2.84~ 2.78 (m, 1H), 2.65~ 2.56 (m, 1H), 2.02~1.91 (m, 2H), 1.53 (s, 9H), 1.08 (t, *J* = 7.5 Hz, 3H). ^13^C NMR (100 MHz, CDCl_3_, 25 *°*C, *mixture of rotamers*) *δ* 168.9, 167.4, 148.8, 148.5, 132.2, 129.9, 122.4, 85.6, 74.5, 55.4, 54.2, 29.3, 28.0, 17.4, 12.9. HRMS (ESI+): m/z calculated for C_19_H_24_ClNO_8_S [M+Na] ^+^ 484.9100, found 484.0796.

**C07c**: 103 mg (86%). white solid. Mp. =112~ 115.1 °C. ^1^H NMR (400 MHz, CDCl_3_, 25 *°*C, *mixture of rotamers*) *δ* 7.63 (d, *J* = 4.1 Hz, 1H), 7.40~ 7.38 (m, 2H), 7.08~ 7.06 (m, 2H), 6.99 (d, *J* = 4.1 Hz, 1H), 5.22 (dd, *J* = 10.0, 8.6 Hz, 1H), 4.87 (dd, *J* = 9.8, 1.6 Hz, 1H), 2.85~ 2.79 (m, 1H), 2.68~ 2.59 (m, 1H), 1.52 (s, 9H). ^13^C NMR (100 MHz, CDCl_3_, 25 *°*C, *mixture of rotamers*) *δ* 169.0, 165.7, 148.8, 148.5, 140.7, 135.0, 132.7, 132.3, 130.0, 127.3, 122.4, 85.6, 75.2, 55.4, 29.3, 28.0. HRMS (ESI+): m/z calculated for C_20_H_19_C_l2_NO_8_S_2_ [M+H] ^+^ 537.3910, found 537.5345.

**C07e**: 110 mg (77%). colorless oil. ^1^H NMR (500 MHz, CDCl_3_, 25 *°*C, *mixture of rotamers*) *δ* 7.87 (d, *J* = 8.3 Hz, 2H), 7.40~ 7.35 (m, 4H), 7.08 ~7.05 (m, 2H), 5.12 (dd, *J* = 10.0, 8.6 Hz, 1H), 4.84 (dd, *J* = 9.9, 1.7 Hz, 1H), 2.83~ 2.78 (m, 1H), 2.66~ 2.59 (dt, *J* = 14.0, 9.9 Hz, 1H), 2.44 (s, 3H), 1.50 (s, 9H). ^13^C NMR (125 MHz, CDCl_3_, 25 *°*C, *mixture of rotamers*) *δ* 169.1, 166.0, 148.9, 148.5, 145.8, 132.5, 132.2, 130.2, 129.9, 128.4, 122.4, 85.4, 74.0, 55.4, 29.5, 28.0, 21.9. HRMS (ESI+): m/z calculated for C_23_H_24_ClNO_8_S [M+H] ^+^ 510.0540, found 511.4706.

**C07f**: 107 mg (76%). slight yellow oil.^1^H NMR (400 MHz, CDCl_3_, 25 *°*C, *mixture of rotamers*) *δ* 8.11~ 7.94 (m, 2H), 7.40~ 7.37 (m, 4H), 7.07 (d, *J* = 8.8 Hz, 2H), 5.23 (dd, *J* = 10.1, 8.6 Hz, 1H), 4.86 (dd, *J* = 9.8, 1.4 Hz, 1H), 2.87~ 2.81 (m, 1H), 2.70~ 2.60 (m, 1H), 1.52 (s, 9H). ^13^C NMR (100 MHz, CDCl_3_, 25 *°*C, *mixture of rotamers*) *δ* 169.0, 166.0, 153.5, 148.8, 148.5, 134.0, 132.3, 130.7, 130.0, 122.4, 121.1, 85.6, 74.6, 55.4, 29.4, 28.4, 28.0, 28.0. HRMS (ESI+): m/z calculated for C_23_H_21_ClF_3_NO_9_S [M+Na] ^+^ 602.0577, found 602.0470.

**C07g**: 116 mg (80%). white solid. Mp. =144~ 145.5 °C.^1^H NMR (400 MHz, CDCl_3_, 25 *°*C, *mixture of rotamers*) *δ* 8.07~ 7.98 (m, 2H), 7.38 (d, *J* = 8.9 Hz, 2H), 7.28~ 7.22 (m, 3H), 7.09~ 7.06 (m, 2H), 5.20 (dd, *J* = 10.0, 8.6 Hz, 1H), 4.86 (dd, *J* = 9.9, 1.5 Hz, 1H), 2.86~ 2.80 (m, 1H), 2.68~ 2.59 (m, 1H), 1.52 (s, 9H). ^13^C NMR (125MHz, CDCl_3_, 25 *°*C, *mixture of rotamers*) *δ* 169.1, 166.1, 148.8, 148.5, 132.3, 131.8, 131.4, 131.3, 129.9, 122.4, 117.0, 116.8, 85. 6, 55.4, 29.4, 28.0, 28.0. HRMS (ESI+): m/z calculated for C_22_H_21_ClFNO_8_S [M+H] ^+^ 513.0660, found 514.1511.

**C07h**: 128 mg (81%). white solid. Mp. =121~ 121.5 °C. ^1^H NMR (400 MHz, CDCl_3_, 25 *°*C, *mixture of rotamers*) *δ* 8.15 (d, *J* = 8.2 Hz, 2H), 7.85 (d, *J* = 8.3 Hz, 2H), 7.40~ 7.37 (m, 2H), 7.09~ 7.05 (m, 2H), 5.27 (dd, *J* = 10.1, 8.6 Hz, 1H), 4.87 (dd, *J* = 9.9, 1.5 Hz, 1H), 2.86~ 2.83 (m, 1H), 2.70~ 2.62 (m, 1H), 1.53 (s, *J* = 14.5 Hz, 9 H). ^13^C NMR (100 MHz, CDCl_3_, 25 *°*C, *mixture of rotamers*) *δ* 168.9, 165.8, 148.7, 148.4, 139.3, 132.2, 129.8, 129.8, 128.8, 126.5, 126.5, 122.3, 85.6, 74.8, 55.3, 29.3, 27.9, 27.9. HRMS (ESI+): m/z calculated for C_23_H_21_ClF_3_NO_8_S for [M+Na] ^+^ 586.0628, found 586.0515.

**C07i**: 121 mg (80%). white solid. Mp. =153~156.8 °C. ^1^H NMR (500 MHz, DMSO-*d_6_*, 25 *°*C, *mixture of rotamers*) *δ* 8.49 (d, *J* = 8.9 Hz, 2H), 8.28 (d, *J* = 8.9 Hz, 2H), 7.55 (d, *J* = 8.9 Hz, 2H), 7.25 (d, *J* = 8.7 Hz, 2H), 5.59 (t, *J* = 9.3 Hz, 1H), 5.01 (dd, *J* = 10.4, 1.5 Hz, 1H), 2.89~ 2.84 (m, 1H), 2.65~ 2.58 (m, 1H), 1.43 (s, 9H). ^13^C NMR (125 MHz, DMSO-*d_6_*, 25 *°*C, *mixture of rotamers*) *δ* 169.3, 166.9, 151.4, 149.1, 148.7, 141.5, 131.2, 130.2, 130.1, 125.5, 123.9, 84.5, 76.9, 55.7, 27.9. HRMS (ESI+): m/z calculated for C_22_H_21_ClN_2_O_10_S [M+Na] ^+^ 563.0605, found 563.5517.

**C07j**:137 mg (82%). slight yellow oil. ^1^H NMR^.^ (500 MHz, DMSO-*d_6_*, 25 *°*C, *mixture of rotamers*) *δ* 8.04 (dd, *J* = 9.8, 1.8 Hz, 1H), 7.89~ 7.86 (m, 1H), 7.74 (dd, *J* = 8.5, 1.8 Hz, 1H), 7.55 ~ 7.53 (m, 2H), 7.26~ 7.23 (m, 2H), 5.49 (t, *J* = 9.3 Hz, 1H), 5.01 (dd, *J* = 10.2, 1.5 Hz, 1H), 2.91~ 2.86 (m, 1H), 2.68~ 2.61 (m, 1H), 1.43 (s, 9H). ^13^C NMR (125 MHz, DMSO-*d_6_*, 25 *°*C, *mixture of rotamers*) *δ* 168.9, 166.4, 148.6, 148.1, 131.9, 130.7, 129.7, 128.7, 123.4, 84.0, 55.3, 27.4. HRMS (ESI+): m/z calculated for C_22_H_20_BrClFNO_8_S [M+Na] ^+^ 613.9765, found 613.1807.

**C07k**: 112 mg (79%). slight yellow oil. ^1^H NMR (500 MHz, CDCl_3_, 25 *°*C, *mixture of rotamers*) *δ* 7.84 (dd, *J* = 3.8, 1.4 Hz, 1H), 7.75 (dd, *J* = 4.0, 1.4 Hz, 1H), 7.40~ 7.37 (m, 2H), 7.17~ 7.16 (m, 1H), 7.00~ 7.06 (m, 2H), 5.20 (dd, *J* = 9.9, 8.6 Hz, 1H), 4.86 (dd, *J* = 9.9, 1.6 Hz, 1H), 2.82~ 2.79 (m, 1H), 2.67~ 2.63 (m, 1H), 1.51 (s, 9H). ^13^C NMR (125 MHz, CDCl_3_, 25 *°*C, *mixture of rotamers*) *δ* 169.1, 165.7, 148.3, 148.1, 135.5, 134.9, 132.2, 129.94 128.0, 122.4, 85.5, 55.4, 29.4, 28.0. HRMS (ESI+): m/z calculated for C_20_H_20_ClNO_8_S_2_ [M+Na] ^+^ 524.9490, found 524.2334.

**C07l**: 124 mg (81%). slight yellow oil. ^1^H NMR (400 MHz, CDCl_3_, 25 *°*C, *mixture of rotamers*) *δ* 8.56 (s, 1H), 8.04~ 7.92 (m, 4H), 7.71~ 7.62 (m, 2H), 7.39~ 7.35 (m, 2H), 7.07~ 7.03 (m, 2H), 5.21 (dd, *J* = 9.9, 8.5 Hz, 1H), 4.85 (dd, *J* = 9.9, 1.7 Hz, 1H), 2.86~ 2.80 (m, 1H), 2.70~ 2.61 (m, 1H), 1.49 (s, 9H). ^13^C NMR (100 MHz, CDCl_3_, 25 *°*C, *mixture of rotamers*) *δ* 169.1, 165.9, 148.9, 148.50 135.7, 132.4, 132.2, 132.0, 130.3, 130.0, 129.9, 129.8, 129.7, 128.2, 128.0, 122.7, 122.41,85.4, 74.3, 55.4, 29.5, 28.0. HRMS (ESI+): m/z calculated for C_26_H_24_ClNO_8_S [M+Na] ^+^ 568.9870, found 568.4342.

**C07m**: 96 mg (75%). colorless oil. ^1^H NMR (500 MHz, CDCl_3_, 25 *°*C, *mixture of rotamers*) *δ* 7.39~ 7.36 (m, 2H), 7.09~ 7.06 (m, 2H), 5.43 (dd, *J* = 10.3, 8.7 Hz, 1H), 4.88 (dd, *J* = 9.8, 1.4 Hz, 1H), 3.83 (d, *J* = 15.2 Hz, 1H), 3.50 (d, *J* = 15.1 Hz, 1H), 2.86~ 2.79 (m, 1H), 2.66~ 2.60 (m, 1H), 2.43~ 2.36 (m, 2H), 2.12 (t, *J* = 4.5 Hz, 1H), 2.09~ 2.02 (m, 1H), 1.96 (d, *J* = 18.6 Hz, 1H), 1.81~ 1.75 (m, 1H), 1.53 (s, 9H), 1.11 (s, 3H), 0.90 (s, 3H). ^13^C NMR (125 MHz, CDCl_3_, 25 *°*C, *mixture of rotamers*) *δ* 214.0, 168.9, 167.3, 148.8, 148.5, 132.2, 129.9, 122.4, 85.5, 74.5, 58.2, 55.5, 49.9, 48.2, 42.9, 42.6, 29.2, 28.0, 27.0, 25.4, 19.8. HRMS (ESI+): m/z calculated for C_25_H_30_ClNO_9_S [M+Na] ^+^ 579.0230, found 578.1573.

**General Procedure**: TFA (1.5 equiv) was added to sulfonyl esters **C07a~m** (1.0 equiv) in CH_2_Cl_2_ (0.2 M) and the mixture was stirred at room temperature for 0.5~2h. The reaction was quenched with saturated NaHCO_3_ aqueous solution and the mixture was separated through a separating funnel. The aqueous phase was extracted by CH_2_Cl_2_ (🞨3), and dried with anhydrous Na_2_SO_4_. After removal solvent by reduce pressure, the crude residue was purified using silica gel flash column chromatography [eluent: EtOAc/Petroleum ether] to give compound **C08a~n**.

**C08a**: 33 mg (84%). white solid. Mp. =132.0~ 135.1 °C. ^1^H NMR (500 MHz, DMSO-*d_6_*) *δ* 8.99 (s, 1H), 7.48 (d, *J* = 8.7 Hz, 2H), 7.24 (d, *J* = 8.7 Hz, 2H), 5.20 (t, *J* = 8.3 Hz, 1H), 4.58 (dt, *J* = 9.6, 1.9 Hz, 1H), 3.72~ 3.68 (m, 1H), 2.92~ 2.87 (m, 1H), 2.58~ 2.53 (m, 1H), 1.32~ 1.29 (m, 6H). ^13^C NMR (125 MHz, DMSO-*d_6_*) *δ* 171.2, 171.0, 156.9, 149. 5, 130.9, 130.1, 130.1, 129.7, 124.2, 117.5, 75.9, 52.5, 52.5, 31.9, 16.8, 16.5. HRMS (ESI+): m/z calculated for C_14_H_16_ClNO_6_S [M+Na] ^+^ 384.0386, found 384.0273.

**C08b**: 26 mg (65%). white solid. Mp. =140.2~ 141.2 °C. ^1^H NMR (500 MHz, DMSO-*d_6_*) *δ* 8.97 (s, 1H), 7.54~ 7.51 (m, 2H), 7.29~ 7.26 (m, 2H), 5.25 (t, *J* = 8.2 Hz, 1H), 4.62 (dt, *J* = 9.6, 1.9 Hz, 1H), 3.52~ 3.43 (m, 2H), 2.96~ 2.91 (m, 1H), 2.62~ 2.57 (m, 1H), 1.85~1.71 (m, 2H), 1.00 (t, *J* = 7.4 Hz, 3H). ^13^C NMR (125 MHz, DMSO-*d_6_*) *δ* 170.8, 170.5, 149.0, 130.5, 129.6, 123.7, 75.3, 52.1, 51.9, 31.3, 16.9, 12.5. HRMS (ESI+): m/z calculated for C_14_H_16_ClNO_6_S [M+Na] ^+^ 384.0386, found 384.1932.

**C08c**: 26 mg (64%). white solid. Mp. =139.4~ 143 °C. ^1^H NMR^.^ (400 MHz, DMSO-*d_6_*) *δ* 9.01 (s, 1H), 7.90 (d, *J* = 4.2 Hz, 1H), 7.53~ 7.51 (m, 2H), 7.43 (d, *J* = 4.2 Hz, 1H), 7.28~7.25 (m, 2H), 5.27 (t, *J* = 8.4 Hz, 1H), 4.63~ 4.58 (m, 1H), 2.84~ 2.78 (m, 1H), 2.57~ 2.53 (m, 1H). ^13^C NMR (125 MHz, DMSO-*d_6_*) *δ* 170.7, 170.0, 156.9, 149.4, 138.74 136.4, 133.4, 130.9, 130.0, 129.7, 129.2, 124.1, 117.5, 77.3, 52.6, 31.2. HRMS (ESI+): m/z calculated for C_15_H_11_Cl_2_NO_6_S_2_ [M+Na] ^+^ 457.9404, found 458.2637.

**C08e**: 60 mg (75%). white solid. Mp. =158~ 158.6 °C. ^1^H NMR (500 MHz, DMSO-*d_6_*) *δ* 8.96 (s, 1H), 7.86 (d, *J* = 8.1 Hz, 2H), 7.52~ 7.49 (m, 4H), 7.26~ 7.22 (m, 2H), 5.12 (t, *J* = 8.0 Hz, 1H), 4.56 (dd, *J* = 10.1, 2.5 Hz, 1H), 2.73~ 2.68 (m, 1H), 2.44~ 2.36 (m, 4H). ^13^C NMR (125 MHz, DMSO-*d_6_*) *δ* 170.3, 169.9, 149.0, 145.4, 132.8, 130.5, 130.3, 129.6, 127.9 123.7, 75.6 52.1, 31.0, 21.2. HRMS (ESI+): m/z calculated for C_18_H_16_ClNO_6_S [M+Na] ^+^ 432.0386, found 432.0273.

**C08f**: 32 mg (77%). white solid. Mp. =150.9~ 151.3 °C. ^1^H NMR (500 MHz, DMSO-*d_6_*) *δ* 8.98 (s, 1H), 8.15~ 8.13 (m, 2H), 7.70~ 7.69 (m, 2H), 7.52~ 7.50 (m, 2H), 7.26~ 7.24 (m, 2H), 5.25 (t, *J* = 7.9 Hz, 1H), 4.59 (dt, *J* = 9.6, 2.0 Hz, 1H), 2.80~ 2.75 (m, 1H), 2.54~ 2.51 (m, 1H). ^13^C NMR (125 MHz, DMSO-*d_6_*) *δ* 170.7 170.3 152.7, 149.4, 135.1, 131.2, 130.9, 130.02, 124.1, 122.3,76.7, 52.6, 31.3. HRMS (ESI+): m/z calculated for C_18_H_13_ClF_3_NO_7_S [M+Na] ^+^ 502.0053, found 501.9970.

**C08g**: 33 mg (81%). white solid. Mp. =155.9~ 156.5 °C. ^1^H NMR (500 MHz, DMSO-*d_6_*) *δ* 8.97 (s, 1H), 8.09~ 8.06 (m, 2H), 7.55 (t, *J* = 8.9 Hz, 2H), 7.53~7.49 (m, 2H), 7.26~ 7.24 (m, 2H), 5.21 (t, *J* = 8.0 Hz, 1H), 4.58 (dt, *J* = 9.6, 2.0 Hz, 1H), 2.77~ 2.72 (m, 1H), 2.48~ 2.43 (m, 1H). ^13^C NMR (125 MHz, DMSO-*d_6_*) *δ* 170.7, 170.3, 166.9, 164.9 156.9 149.4, 131.7, 131.6, 130.9, 130.0, 129.7, 124.1, 117.7, 117.5, 117. 5, 76.4, 52.6, 31.3. HRMS (ESI+): m/z calculated for C_17_H_13_ClFNO_6_S [M+Na] ^+^ 436.0136, found 435.9789.

**C08h**: 32 mg (78%). white solid. Mp. =164.6~ 165.5 °C. ^1^H NMR (400 MHz, DMSO-*d_6_*) *δ* 8.99 (s, 1H), 8.22 (d, *J* = 8.2 Hz, 3H), 8.09 (d, *J* = 8.5 Hz, 3H), 7.52~ 7.50 (m, 2H), 7.26~ 7.24 (m, 2H), 5.30 (t, *J* = 8.4 Hz, 1H), 4.60 (dd, *J* = 9.6, 2.4, 1.3 Hz, 1H), 2.80 (dd, *J* = 13.9, 8.5, 2.4 Hz, 1H), 2.58 – 2.51 (m, 1H). ^13^C NMR (100 MHz, DMSO-*d_6_*) *δ* 170.6 170.1, 149.4, 140.2,130.8, 129.9, 129.4,127.4, 127.4, 126.8, 124.1, 77.0, 52.5, 31.2. HRMS (ESI+): m/z calculated for C_18_H_13_ClF_3_NO_6_S [M+Na] ^+^ 486.0104, found 487.9684.

**C08i**: 32 mg (80%). white solid. Mp. =164.4~ 166.3 °C. ^1^H NMR (500 MHz, DMSO-*d_6_*) *δ* 8.50~ 8.48 (m, 4H), 8.25~ 8.19 (m, 3H), 8.21~ 8.19 (m, 1H), 7.84~ 7.81 (m, 1H), 5.25~ 5.23 (m, 1H), 5.03 (dd, *J* = 9.1, 5.0 Hz, 1H), 2.74~ 2.68 (m, 1H), 2.37~ 2.31 (m, 1H). ^13^C NMR (125 MHz, CDCl_3_) *δ* 168.9, 166.0, 141.6, 130.0, 129.8, 124.6, 122.4, 85.8, 75.4, 55.4, 28.0. HRMS (ESI+): m/z calculated for C_17_H_13_ClN_2_O_8_S [M+Na] ^+^ 463.8070, found 463.9891.

**C08j**: 26 mg (64%). white solid. ^1^H NMR (500 MHz, DMSO-*d_6_*) *δ* 8.05 (s, 1H), 8.02 ~ 7.98 (m, 1H), 7.55 (t, *J* = 8.0 Hz, 1H), 7.35 (d, *J* = 8.6 Hz, 1H), 7.27~ 7.22 (m, 2H), 7.13~7.10 (m, 2H), 5.25 (d, J = 19.3 Hz, 1H), 4.49 (d, *J* = 22.4 Hz, 1H), 2.54~2.52 (m, 1H), 2.48~ 2.46 (m, 1H). HRMS (ESI+): m/z calculated for C_17_H_12_BrClFNO_6_S [M+Na] ^+^ 513.9241, found 514.3247.

**C08k**: 30 mg (75%). white solid. Mp. =141.7~ 142.1 °C. ^1^H NMR (400 MHz, DMSO-*d_6_*) *δ* 9.03 (s, 1H), 8.21 (dd, *J* = 4.0, 1.4 Hz, 1H), 7.97 (dd, *J* =4.0, 1.4 Hz, 1H), 7.52~ 7.50 (m, 2H), 7.33~ 7.32 (m, 1H), 7.26~ 7.24 (m, 2H), 5.20 (t, *J* = 8.4 Hz, 1H), 4.59 (dt, *J* = 9.6, 2.5 Hz, 1H), 2.76~ 2.70 (m, 1H), 2.49 – 2.42 (m, 1H). ^13^C NMR (100 MHz, DMSO-*d_6_*) *δ* 170.6, 170.1, 149.4, 136.9, 136.2, 135.1, 130.8, 129.5, 128.8, 124.1, 76.7, 52.5, 31.1. HRMS (ESI+): m/z calculated for C_15_H_12_ClNO_6_S_2_ [M+Na] ^+^ 423.8320, found 424.3030.

**C08l**: 60 mg (75%). white solid. ^1^H NMR (500 MHz, DMSO-*d_6_*) *δ* 8.95 (s, 1H), 8.73 (d, *J* = 2.1 Hz, 1H), 8.24 (t, *J* = 8.2 Hz, 2H), 8.11 (d, *J* = 8.1 Hz, 1H), 7.94 (dd, *J* = 8.7, 2.0 Hz, 1H), 7.79~ 7.77 (m, 1H), 7.75~ 7.73 (m, 1H), 7.50~ 7.48 (m, 2H), 7.22~ 7.20 (m, 2H), 5.22 (t, *J* = 8.0 Hz, 1H), 4.56 (dt, *J* = 9.5, 2.1 Hz, 1H), 2.73~ 2.68 (m, 1H), 2.48~ 2.42 (m, 1H). ^13^C NMR (125 MHz, DMSO-*d_6_*) *δ* 170.2, 169.7, 148.9, 135.0, 132.6, 131.6, 130.4, 130.0, 129.9, 129.8, 129.7, 129.5, 129.2, 128.1, 128.1, 123.6, 122.43, 116.9, 75.8, 52.1, 30.9. HRMS (ESI+): m/z calculated for C_21_H_16_ClNO_6_S [M+Na] ^+^ 468.9700, found 468.0036.

**C08m**: 35 mg (85%). white solid. Mp. =141.1~ 143.9 °C. ^1^H NMR (400 MHz, DMSO-*d_6_*) *δ* 9.70 (s, 1H), 7.21~ 7.17 (m, 2H), 6.78~ 6.74 (m, 2H), 5.29 (dd, *J* = 8.8, 5.9 Hz, 1H), 5.11 (dd, *J* = 9.1, 4.7 Hz, 1H), 3.62 (d, *J* = 15.1 Hz, 1H), 3.53 (d, *J* = 15.2 Hz, 1H), 2.98~ 2.83 (m, 1H), 2.43~ 2.19 (m, 2H), 2.09 (t, *J* = 4.4 Hz, 1H), 1.99~ 1.94 (m, 2H), 1.59~ 1.564 (m, 1H), 1.46~ 1.43 (m, 1H), 1.04 (s, 3H), 0.83 (s, 3H). ^13^C NMR (125 MHz, CDCl_3_) *δ* 214.0, 168.9, 167.3, 148.8, 148.5, 132.17 129.90 129.7, 122.8, 122.43, 85.4, 74.4, 58.2, 55.5, 49.8, 48.2, 42.8, 42.6, 29.2, 27.0, 25.4, 19.8. HRMS (ESI+): m/z calculated for C_20_H_22_ClNO_7_S [M+Na] ^+^ 478.0805, found 478.7579.

**C08n**: 26 mg (75%). white solid. Mp. =139~ 142.6 °C. ^1^H NMR (400 MHz, DMSO-*d_6_*) *δ* 8.92 (s, ****1H), 7.54~ 7.52 (m, 2H), 7.23~ 7.21 (m, 2H), 4.61 (dt, *J* = 7.7, 2.9, 1.3 Hz, 2H), 3.15~ 3.07 (m, 1H), 2.48~ 2.46 (m, 1H). ^13^C NMR (125 MHz, DMSO-*d_6_*) *δ* 156.4, 129.6, 129.2, 123.6, 122.4, 117.0, 67.0, 66.9, 51.8, 31.8. HRMS (ESI+): m/z calculated for C_11_H_10_ClNO_4_ [M+Na] ^+^ 278.0298, found 278.2456.

**Supplementary Results**

Figures. S1. The ^1^H NMR and ^13^C NMR spectra of intermediates and target compounds
